# Nutritional composition and glycemic index analyses of vitamin A‐biofortified maize in healthy subjects

**DOI:** 10.1002/fsn3.801

**Published:** 2018-10-08

**Authors:** Olarewaju M. Oluba, Ajoke B. Oredokun‐Lache

**Affiliations:** ^1^ Food Safety and Toxicology Research Unit Department of Biological Sciences Environment and Technology Research Cluster College of Science and Engineering Landmark University Omu Aran Nigeria; ^2^ Food and Nutrition Science Laboratory International Institute of Tropical Agriculture Ibadan Nigeria

**Keywords:** dietary fibers, glycemic index, healthy subjects, pro‐vitamin A carotenoids, vitamin A‐biofortified maize

## Abstract

Besides being a veritable tool for easing the problem of vitamin A deficiency (VAD), this study sought to explore another potential health benefit of vitamin A‐biofortified maize (VABM). In the present study, the nutritional composition and glycemic index (GI) of tuwo masara (a nonfermented maize‐based dumpling), made from VABM and the indigenous white maize (IWM) genotype, were evaluated. VABM showed significantly (*p *<* *0.05) lower fat (4.38 ± 0.46%) and crude protein (6.58 ± 0.13%) but higher crude fiber (5.29 ± 0.0%) contents compared to 5.22 ± 0.25% crude fat, 7.28 ± 0.11% crude protein, and 4.69 ± 0.00% crude fiber in the IWM. The phytic acid content in the IWM (2.77 mg/100 g) was 39% higher than the level (2.0 ± 0.04 mg/100 g) in VABM. The major provitamin A carotenoid in the VABM were lutein (7.37 ± 0.52 μg/g), zeaxanthin (1.65 ± 0.01 μg/g), cryptoxanthin (1.29 ± 0.02 μg/g), and all‐trans‐β‐carotene (0.83 ± 0.02 μg/g), while the IWM contained only lutein (1.52 ± 0.32 μg/g). The total carotene concentration, 12.74 ± 1.13 μg/g dry weight in the VABM, was over eight times higher than that observed for the IWM, 1.52 ± 0.32 μg/g dry weight. The VABM tuwo masara showed a significantly lower GI value (70.3%) compared to the IWM tuwo masara (87.7%). Data obtained from the study further attest to the positive nutritional and health benefits of VABM.

## 1 INTRODUCTION

Vitamin A deficiency (VAD) is a major public health concern among preschool children and women in developing countries (Arlappa et al., 2011; Bowley, 2008). Consequently, an estimated average of one out of every three preschool children and one quarter of pregnant women in Nigeria are vitamin A deficient (Maziya‐Dixon et al., 2006). Therefore, several intervention strategies have been posited to address the devastating consequences of VAD on children (Bouis, Holz, McClafferty, Meenakshi, & Pfeiffer, 2011).

Using traditional plant‐breeding and bioengineering strategies, high provitamin A, yellow maize genotypes have been genetically modified to accumulate high levels of pro‐vitamin A and other carotenoids, as well as other micronutrients (Ortiz‐Monasterio et al., 2007). Since its introduction in 2005, the seeds of this improved maize variety are continuously being distributed to local farmers who in turn plant and share these seeds over generations as a cost‐effective, renewable means to reduce micronutrient deficiencies (Pfeiffer & McClafferty, 2007). Consequently, it is expected that enhanced provitamin A contents in the maize kernel will have a far‐reaching health impact in Africa given its level of consumption by the different strata of the society, including children and women who are most susceptible to VAD (Bouis et al., 2011).

Tuwo masara (a non‐fermented maize‐based dumpling) is a popular food product made from maize in Nigeria. It is consumed in the northern part of Nigeria and other Hausa‐speaking West African communities (Boladale, Usman, Rasheed, Benson, & Salifou, 2002). It is a gel‐like food product made from a combination of maize flour, water, and heat (Boladale & Adeyemi, 2014). It is eaten with different type of soups, including vegetable, okra, or ogbono soup. In spite of its nutritional and health‐promoting potentials, the current trend in terms of acceptability and production of vitamin A‐biofortified maize (VABM) is low when compared with the indigenous white maize (IWM) varieties in Nigeria. This could be attributed to the low level of awareness of the nutritional attributes of these new maize varieties among the masses. Thus, in addition to the current efforts at promoting the cultivation and consumption of VABM through aggressive extension services, experimental research aimed at validating the acclaimed nutritional potential of these new maize varieties could further provide a boost to the current level of its consumer acceptability. Hence, this study was aimed at carrying out a comparative nutritional analysis in terms of proximate composition, antinutritional composition, and total carotenoid level between maize flour made from VABM and IWM varieties, and, in addition, evaluates the glycemic index of tuwo masara made from these maize flour samples, respectively, in healthy subjects.

## 2 MATERIALS AND METHODS

### 2.1 Plant materials

The seeds of two maize varieties, TZL COMP4 C2 (IWM) and BRY 9928 DMR SR (VABM), were obtained from the Crop Multiplication Unit of the International Institute of Tropical Agriculture (IITA), Ibadan, Nigeria, and authenticated by Mr. Phil M. Lache (a Research Technician at IITA) and Mr. Kehinde Oyebanji, a taxonomist in the Department of Crop Science, Joseph Ayo Babalola University, Ikeji Arakeji, Osun State, Nigeria.

### 2.2 Nutritional composition analyses

#### 2.2.1 Proximate composition

Proximate composition analysis of the respective flour of the two maize varieties (VABM and IWM) was analyzed following standard methods (AOAC, 1995). Percentage amylose, sugar, and starch contents were determined following the procedures of Juliano et al. (1981).

#### 2.2.2 Anti‐nutritional factor

Phytate, tannin, and cyanogenic potential levels in the respective maize flour were determined using the method of AOAC (1995). The colorimetric method of Vaintraub and Lapteva (1988) and modified by Gao et al. (2007) was employed in the quantification of the phytate content using phytic acid solution to obtain the standard curve. The total polyphenols content and tannins were determined by the method of Marigo (1973). The optical densities were read at 725 nm. Total dietary fiber content was estimated using the enzymatic‐gravimetric method described by Asp, Johansson, Hallmer, and Siljestom (1983).

#### 2.2.3 Total carotenoids

Carotenoid extraction was done according to the procedure previously described by Rodriguez‐Amaya and Kimura (2004) with little modifications and the individual carotenoid quantified against a calibration curve obtained using standard carotenoids based on the observed peak areas.

### 2.3 Preparation of tuwo masara

Tuwo masara (i.e., maize tuwo) was prepared according to the procedure previously described by Boladale et al. (2002). The tuwo was prepared from dried maize in which the outer coat (testa) has been removed by grinding gently inside mortar with pestle. A small quantity of water was added to the grains to facilitate the testa removal process. Thereafter, the grains in which the testa has been removed were sun‐dried, milled using a mechanical grinder to obtain smooth, whitish flour. The flour was sieved and added to hot boiling water with continuous stirring to form a thickened gel‐like paste herewith referred to as “tuwo masara.”

### 2.4 Glycemic index

#### 2.4.1 Study population

The study was undertaken at Joseph Ayo Babalola University, Ikeji Arakeji (Nigeria), a fully residential private University. Sixty‐five apparently healthy, nondiabetic human subjects (23 males and 42 females) aged between 17 and 39 years were recruited for the feeding trial upon obtaining their informed consent.

#### 2.4.2 Glycemic index analysis

The glycemic index (GI) was calculated according to the formula described by Jenkins et al. (1981) as follows:

$$\text{GI}\;\left(\%\right)=\frac{\begin{array}{cc} & \text{Incremental area under the 2‐h glucose response curve}\;\\ & \text{for a 50‐g carbohydrate equivalent of the test food} \end{array}}{\begin{array}{cc} & \text{Incremental area under the 2‐h glucose}\;\\ & \text{response curve for a 50‐g glucose} \end{array}}$$

### 2.5 Statistical analysis

Data are expressed as mean ± *SD* or *SEM* as applicable. Statistical comparisons between samples/subjects were made by paired Student's *t* tests or one‐way ANOVA as applicable. The level of statistical significance was set at *p* < 0.05.

## 3 RESULTS

### 3.1 Proximate composition

The proximate composition profile for the VABM and IWM used in this study as presented in Table 1 showed a nonsignificant difference in terms of moisture (8.66 ± 0.25%, 8.33 ± 0.23%) and ash (1.23 ± 0.11%, 1.34 ± 0.04%), respectively. However, percentage fat and crude protein contents were significantly higher in the IWM compared to the VABM. Crude fiber content was significantly lower in the IWM (4.69 ± 0.00%) compared to the VABM (5.29 ± 00%). There was no significant variation in percentage total carbohydrate, sugar, starch, amylose, and amylopectin contents in the two maize genotypes. In addition, the percentage amylose‐to‐amylopectin ratio was not significantly different in the two maize samples.

**Table 1:** Proximate composition (%) of indigenous white maize (IWM) and vitamin A‐biofortified yellow maize (VABM)

Proximate composition	Indigenous white maize (TZL COMP4 C2)	Vitamin A‐biofortified yellow maize (BRY 9928 DMR SR)
Moisture	8.66 ± 0.25^a^	8.33 ± 0.23^a^
Ash	1.23 ± 0.11^a^	1.34 ± 0.04^a^
Fat	5.22 ± 0.25^b^	4.38 ± 0.46^a^
Crude protein	7.28 ± 0.11^b^	6.58 ± 0.13^a^
Crude fiber	4.69 ± 0.00^a^	5.29 ± 0.0^b^
Total carbohydrate	72.89 ± 0.49^a^	74.07 ± 0.06^a^
Sugar	7.23 ± 0.13^a^	7.56 ± 0.18^a^
Starch	88.28 ± 1.16^a^	88.96 ± 1.35^a^
Amylose	14.36 ± 0.13^a^	14.17 ± 0.13^a^
Amylopectin	73.90 ± 2.52^a^	74.8 ± 5.31^a^
Amylose/amylopectin	0.194^a^	0.189^a^

*Note*

Values are means ± *SD* of three determinations. Mean with different superscript letters in column are significantly different (*p *<* *0.05).

### 3.2 Anti‐nutritional factors

The anti‐nutritional profile of the IWM and VABM is shown in Table 2. The results showed that the phytate concentration in VABM (2.0 ± 0.04 mg/100 g) was significantly lower compared with the value recorded for IWM sample (2.77 ± 0.09 mg/100 g). The observed total polyphenolic content and cyanide cyanogenic potential level in the two maize samples were not statistically significant. Total dietary fiber content in the VABM was significantly higher (9.62 ± 0.12) compared to the level in the IWM (6.80 ± 0.25).

**Table 2:** Antinutritional factors of the indigenous white maize (IWM) and vitamin A‐biofortified yellow maize (VABM)

Antinutritic factor	IWM (TZL COMP4 C2) (mg/100 g)	VABM (BRY 9928 DMR SR) (mg/100 g)
Phytate	2.77 ± 09^b^	2.0 ± 0.04^a^
Tannin	1.33 ± 0.03^a^	1.43 ± 0.02^a^
Cyanogenic potential	1.63 ± 0.12^a^	1.71 ± 0.13^a^

*Note*

Values are means ± *SD* of three determinations. Mean with different superscript letters in column are significantly different (*p *<* *0.05).

### 3.3 Total carotenoids

**Figure 1:**
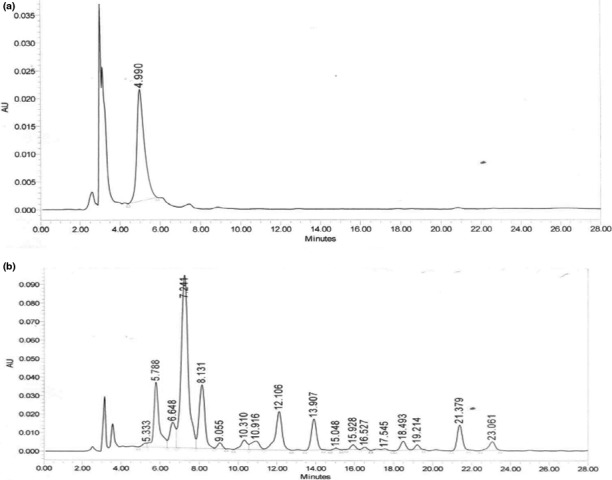
(a) HPLC chromatogram showing the total carotenoid array of the indigenous white maize flour (TZL COMP4 C2 genotype). (b) HPLC chromatogram showing the total carotenoid array of the vitamin A‐biofortified yellow maize flour (BRY 9928 DMR SR genotype)

The carotenoid content of the two maize samples is shown in Table 3. The major provitamin A carotenoids shown in the VABM were lutein (7.37 ± 0.52 μg/g), zeaxanthin (1.65 ± 0.01 μg/g), cryptoxanthin (1.29 ± 0.02 μg/g), and all‐trans‐β‐carotene (0.83 ± 0.02 μg/g). The IWM genotype showed the presence of only lutein (1.52 ± 0.32 μg/g). The total carotene concentration, 12.74 ± 1.13 μg/g dry weight in the VABM, was over eight times higher than that observed for the IWM, 1.52 ± 0.32 μg/g dry weight. The chromatogram showing the carotenoid arrays for the IWM and VABM samples is shown in Figure 1a,b, respectively.

**Table 3:** Carotenoid composition of the indigenous white maize (IWM) and vitamin A‐biofortified yellow maize (VABM)

	Indigenous white maize (TZL COMP4 C2)	Vitamin A‐biofortified yellow maize (BRY 9928 DMR SR)
β‐Cryptoxanthin	1.29 ± 0.02	–
α‐Carotene	0.72 ± 0.00	–
13‐cis‐β‐carotene	0.04 ± 0.00	–
9‐cis‐β‐carotene	0.84 ± 0.00	–
All‐trans‐β‐carotene	0.83 ± 0.02	–
Lutein	7.37 ± 0.52^b^	1.52 ± 0.32^a^
Zeaxanthin	1.65 ± 0.01	–

*Note*

Values are means ± *SD* of three determinations. Mean with different superscript letters in column are significantly different (*p *<* *0.05).

### 3.4 Subjects characteristics

Sixty‐two individuals among staff and students of Joseph Ayo Babalola University, Ikeji Arakeji, Nigeria, volunteered to participate in the study. Every participant completed questionnaires regarding age, carbohydrate metabolism deficiencies, smoking habits, the carbohydrate source, physical activity, and medical history. In addition, information on weight, height, and waist and hip circumferences was obtained. Forty‐five subjects (17 males and 28 females) aged 20.3 ± 5.2 years, mean body mass index (BMI) of 23.3 ± 1.4 kg/m2, mean systolic and diastolic blood pressure of 101.1 ± 12.2 and 78.0 mmHg, respectively, and a mean fasting plasma glucose of 72.1 ± 10.5 mg/dl met the inclusion criteria and participated in the remaining parts of the study.

### 3.5 Glycemic index

**Figure 2:**
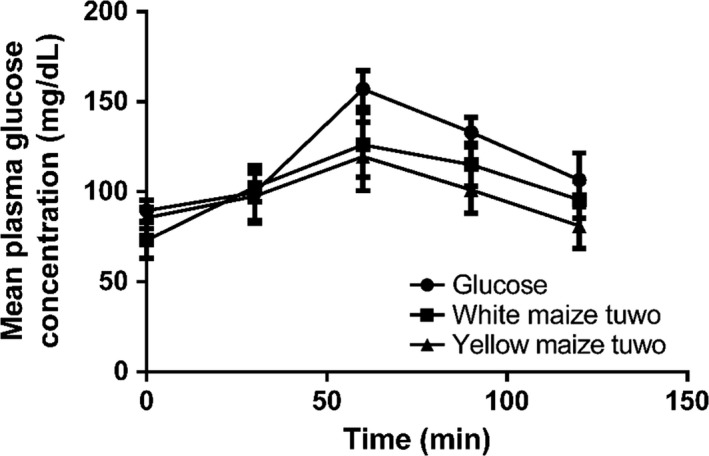
Mean glycemic responses of subjects fed 50 g portion of tuwo prepared with the indigenous white maize (white maize tuwo) and the vitamin A‐biofortified yellow maize (yellow maize tuwo)

The mean glycemic responses to glucose solution and the test foods are shown in Figure 1. The blood glucose response curve to glucose solution and tuwo masara prepared with the IWM and VABM genotypes peaked at 60 min. The postprandial blood glucose responses to the IWM tuwo were higher compared to the VABM tuwo. The peak postprandial plasma glucose (PPPG) for both the IWM and the VABM tuwo was not significantly (*p* > 0.05) different from each other (Table 4). However, the maximum increase in plasma glucose (MIPG) responses for the VABM tuwo was significantly (*p* < 0.05) lower compared to the IWM tuwo. Similarly, the 2‐h postprandial plasma glucose (2hPG) for the VABM tuwo was significantly (*p* < 0.05) lower compared to that for IWM tuwo. The calculated glycemic index (GI) for the VABM tuwo (70.3%) was significantly lower (*p* < 0.05) compared to GI for the IWM tuwo (87.7%) (Figure 2). However, the GIs for tuwo from both IWM and VABM were significantly (*p* < 0.05) lower compared to that of glucose.

**Table 4:** Plasma glucose response indices following consumption of indigenous white maize (IWM) tuwo and vitamin A‐biofortified yellow maize (VABM) tuwo

Test item	*N*	PPPG	MIPG	2hPG	IAUGC	GI (%)
		(mg/dl)	(mg/dl)	(mg/dl)		
Glucose	15	157.0 ± 10.2^b^	67 ± 11.0^c^	106.5 ± 10.6^c^	14,625 ± 110.3^b^	100.0 ± 1.2^b^
IWM tuwo	15	126.0 ± 15.5^a^	53.0 ± 5.8^b^	95.5 ± 8.7^b^	12,833 ± 132.0^a^	87.7 ± 8.5^a^
VABM tuwo	15	119.5 ± 18.1^a^	38.0 ± 6.0^a^	81 ± 15.2^a^	12,038 ± 158.5^a^	82.3 ± 11.8^a^

*Note*

GI: glycemic index; IAUGC: incremental area under the 120‐min plasma glucose curve; MIPG: maximum increase in plasma glucose; *N*: Number of subjects who consumed the index food; PPPG: peak postprandial plasma glucose; 2hPG: 2‐h postprandial glucose.

## 4 DISCUSSION

The data obtained in this study showed that the VABM flour is lower in crude fat, crude protein, and phytic acid but higher in fiber compared to the IWM. In addition, the VABM contained the major provitamin A carotenoids including β‐cryptoxanthin, 13‐cis‐β‐carotene, 9‐cis‐β‐carotene, and all‐trans‐β‐carotene unlike the IWM which is devoid of the major provitamin A carotenoids but for a low amount of zeaxanthin and β‐cryptoxanthin. The GI of the VABM tuwo masara was observed to be lower compared to the IWM tuwo masara.

The two maize varieties analyzed in this study have similar starch, amylose, and amylopectin contents; hence, other factors are presumed to be responsible for the observed difference in their GI. However, the high percentage of the starch in the amylopectin fraction of the two maize varieties is posited to have contributed significantly to their high GI values. Structurally, amylose is composed of several repeated glucose units held in straight chain in α (1–4) glycosidic bonds. These bonds are hydrolyzed by the enzymes maltase and amylase. Amylopectin, on the other hand, contains the same chains, but also has branched points created by α (1–6) glycosidic bonds. The α (1–6) glycosidic linkages in amylopectin are hydrolyzed by the enzyme isomaltase (El‐Harith, Dickerson, & Walker, 1976; Riley et al., 2004). Theoretically, amylose should be easier to digest due to the absence of branched points within its structure and so only one enzyme, amylase, is required for its hydrolysis. However, amylose often forms a very compact physical structure, which inhibits digestion (El‐Harith et al., 1976; Riley et al., 2004). Plasma glucose response has been reported to significantly decrease following consumption of high‐amylose but low‐amylopectin starch diet (Juliano & Goddard, 1986). Earlier investigations by Goddard, Young, and Marcus (1984) showed that plasma glucose response was significantly lower when a high‐amylose rice (Labelle variety, 24% amylose) was fed compared to sweet rice (Mochigome variety, 1% amylose) or glucola drinks.

The presence of antinutritional factors in the maize samples in this study is of significance due to their reported negative health impacts on humans and animals. Experimental evidences from animal studies have shown that phytic acid, when present in plant food, forms complexes with dietary essential minerals such as calcium, zinc, iron, and magnesium and makes them biologically unavailable for absorption (Cheryan & Rackis, 1980; Zhou & Erdman, 1995). High concentration of phytic acid in food has been reported to induce hypocalcemia (Checke & Shull, 1985). The phytic acid level in the two maize genotypes used in the present study is low and well below the acceptable range for seeds and legumes. Studies have shown that the glycemic response of foods varies inversely with the level of phytic acid in the food (Yoon, Thompson, & Jenkins, 1983). The observed total polyphenol content in both the IWM and VABM in this study (1.33–1.43 mg/100 g) was found to be relatively low in comparison with levels found in some literature (Akinyede, Amoo, & Eleyinmi, 2005; Enujiugha, 2003; Umoren, Essien, Ukorebi, & Essien, 2005). The presence of polyphenols in plant foods has been reported to interfere with the activities of some starch‐digesting enzymes such as glucosidases and maltase (Hanhineva et al., 2010). A number of intervention studies have reported considerable improvement in glycemic status in both rats and humans following supplementation with polyphenol‐rich diets (Abesundara, Matsui, & Matsumoto, 2004; Almoosawi, Tsang, Ostertag, Fyfe, & Al‐Dujaili, 2012). The HCl released as a by‐product in the metabolism of cyanogenic glycosides has been implicated in central nervous system dysfunction, respiratory failure, and cardiac arrest (D'Mello, 2000). In addition, the presence of cyanogenic glycosides in animal feeds has also been shown to reduce the amount of metabolizable energy in such feeds (Kumar, 1992). The cyanide content of the two maize varieties used in this study is low and falls within the range considered safe for both humans and other animals.

The data obtained from this study showed that the IWM is devoid of β‐carotene but contained a significantly low amount of zeaxanthin and β‐cryptoxanthin compared to the vitamin A‐biofortified yellow maize genotype. On the other hand, the VABM contained the major provitamin A carotenoids including β‐cryptoxanthin, 13‐cis‐β‐carotene, 9‐cis‐β‐carotene, and all‐trans‐β‐carotene. The total provitamin A carotenoid in the VABM maize reported in this study though higher than the earlier reported range of value (0.25–2.5 μg/g dry weight) reported for a typical yellow maize variety (Berardo, Mazzinelli, Valotti, Lagianna, & Redaelli, 2009; Nuss & Tanumihardjo, 2010) falls short of the expected breeding target of 15 μg/g dry weight for biofortified maize by HarvestPlus (Ortiz‐Monasterio et al., 2007). Naturally, maize has been reported to exhibit variation in their total carotenoid content, with some genotypes reported to contain total carotenoids level as high as 80 μg total carotenoids/g dry weight (Menkir, Liu, White, Maziya‐Dixon, & Rocheford, 2008). The fraction of provitamin A carotenoid of the total carotenoid is usually between 10% and 20%; however, zeaxanthin and lutein each most time represent 30%–50% of total carotenoids in maize (Ortiz‐Monasterio et al., 2007). However, majority of the yellow maize varieties grown and consumed throughout the world contain less than 2 μg provitamin A carotenoids/g dry weight. Vitamin A protects the body against diet‐related chronic diseases as well as reduces the body's predisposition to cataracts, age‐related macular degeneration, and other degenerative diseases (Bertram, 1999; Johnson, 2002).

The observed GI value calculated for the test foods in the present study derives merits in terms of the number of subjects in each group (15), the subjects’ mean age (20.3 ± 5.2 years), their mean BMI (23.3 ± 1.4 kg/m^2^) which were within the WHO recommended normal range, and the subjects’ mean fasting plasma glucose concentration (72.1 ± 10.5 mg/dl) which situates well within the expected normal range of 70–99 mg/dl (Franz, 2004). Although varying factors such as food particle size, methods of preparation, nature of starch, and the type and amount of antinutrient present have been shown to contribute to the glycemic index of foods (Bahado‐Singh, Riley, Wheatley, & Lowe, 2011; Thorne, Thompson, & Jenkins, 1983), more often than not, these factors play little or no role in the physiological properties of food (Bahado‐Singh et al., 2011). The two maize varieties (VABM and IWM) from which the tuwo used in this study was prepared presented similar proximate and antinutritional factors compositions but for the higher fiber and lower phytic acid level noted for the VABM as against the IWM. Thus, the varying dietary fiber content between the VABM and IWM maize genotypes as observed in this study could provide a possible explanation for the differences in the GI of their respective tuwo food products.

Evidence from experimental studies showed that enhanced intake of dietary fibers effectively neutralized the abnormal increase in blood glucose level following a high‐carbohydrate diet (Wenk, 2001). Several mechanisms have been proposed to explain the hypoglycemic action of dietary fibers. Dietary fibers may slow the rate of transit of sugars from the stomach to the small intestine, thus working against a rapid increase in blood glucose concentration following sugar ingestion (Schulze et al., 2004). Increased consumption of fibers has been shown to effectively flatten the sudden surge in blood glucose level following a meal (Salmeron et al., 1997; Schulze et al., 2004). Thus, the high content of total dietary fibers observed in the VABM in this study could be of significance in blood glucose control both in healthy and diabetic subjects.

From the foregoing, the lower GI value observed for the VABM tuwo as against tuwo made from the IWM in this study could be attributed to their varying contents of fiber, phytic acid, and provitamin A carotenoids. Reports from previous experimental studies have shown that increased consumption of carotenoid resulted in significant reduction in plasma glucose concentration. Ford, Will, Bowman, and Narayan (1999) in a population‐based US third National Health and Nutrition Examination Survey observed that serum total carotenoids exhibited an inverse relationship with serum glucose as well as insulin concentrations. In another separate study by Facchini, Humphreys, Donascimento, Abbasi, and Reaven (2000), serum carotenoid concentrations were observed to negatively correlate with insulin resistance and blood glucose concentrations. Furthermore, increased carotenoid intake has been reported to lower the risk of chronic diseases as well as cardiovascular diseases (Bertram, 1999; Fawzi et al., 2000; Johnson, 2002).

## 5 CONCLUSION

Based on the GI value obtained in this study, the tuwo made with VABM could be considered a moderate glycemic index product, based on Englyst and Hudson (1996) classification. Thus, in addition to being a veritable tool for alleviating VAD, the VABM could be considered an alternative energy source for subjects undergoing weight reduction and glycemic control.

## CONFLICT OF INTEREST

The authors declare that they do not have any conflict of interest.

## ETHICAL APPROVAL

Approval (JABUHE002/2015) for the glycemic index study protocol was granted by the Research and Ethics Committee, Joseph Ayo Babalola University, Ikeji Arakeji, Nigeria, and was carried out in compliance with the tenets of the Helsinki Declaration.
